# Recurrent Sleep Restriction During Aging Induces Early Recognition Memory Impairment, Neuroinflammation, and Blood–Brain Barrier Dysfunction in Female Wistar Rats

**DOI:** 10.1007/s11064-026-04909-1

**Published:** 2026-10-08

**Authors:** Ana B. Ramírez-López, Jessica J. Avilez-Avilez, Beatriz Gómez-González, Verónica Salas-Venegas, Ricardo Jair Ramírez-Carreto, Mara A. Guzmán-Ruiz, Anahí Chavarria, Mina Konigsberg

**Affiliations:** 1 Programa de Posgrado en Biología Experimental, Universidad Autónoma Metropolitana, Unidad Iztapalapa, Mexico City, Mexico; 2 Área de Neurociencias, Dpto. Biología de la Reproducción, CBS, Universidad Autónoma Metropolitana, Unidad Iztapalapa, Ciudad de México, Mexico; 3 Unidad de Investigación en Medicina Experimental, Facultad de Medicina, Universidad Nacional Autónoma de México, Mexico City, Mexico; 4 Programa de Doctorado en Ciencias Biomédicas, Universidad Nacional Autónoma de México, Circuito Interior, Av. Universidad 3000, Ciudad Universitaria, Coyoacán, C.P. 04510 Mexico City, Mexico; 5 Departamento de Fisiología, Facultad de Medicina, Universidad Nacional Autónoma de México, Mexico City, Mexico; 6 Departamento de Ciencias Biológicas y de la Salud, Universidad Autónoma Metropolitana, Unidad Iztapalapa, Ciudad de México, Mexico; 7 Departamento de Ciencias de la Salud, División de Ciencias Biológicas y de la Salud, Universidad Autónoma Metropolitana- Iztapalapa, C.P. 09340 Mexico City, Mexico

**Keywords:** Declarative memory; aging, Female rats, Sleep restriction

## Abstract

Sleep restriction is associated with neuroinflammation, blood-brain barrier (BBB) dysfunction, and cognitive impairment. Although reduced sleep duration in women is well recognized and the impact of sleep disorders is correlated with a higher incidence of neurodegenerative diseases, its consequences during female aging remain poorly understood. Hence, the aim of this study was to evaluate whether recurrent sleep restriction in female rats accelerates aging-related processes, compared with aged females with normal sleep patterns. Female Wistar rats were subjected to sleep restriction for 10 consecutive days, every three months from 6 to 18 months of age. An additional group of 3-month-old rats underwent a single sleep restriction episode as control. BBB permeability, neuroinflammation, cellular senescence, and recognition memory were assessed. Recurrent sleep restriction increased BBB permeability in the cerebral cortex to tracers of different molecular weights and induced hippocampal increase in TNF-α and IL-1β. In contrast, senescence markers showed age- and region-dependent changes, with no clear effect of recurrent sleep restriction in aged animals. A single sleep restriction episode was sufficient to impair recognition memory, whereas recurrent sleep restriction accelerated age-related cognitive decline. These findings indicate that the BBB dysfunction and neuroinflammatory alterations during sleep restriction depend on age and brain region, as well as the recurrence of these episodes.

**Graphical Abstract:**

**Figure:**
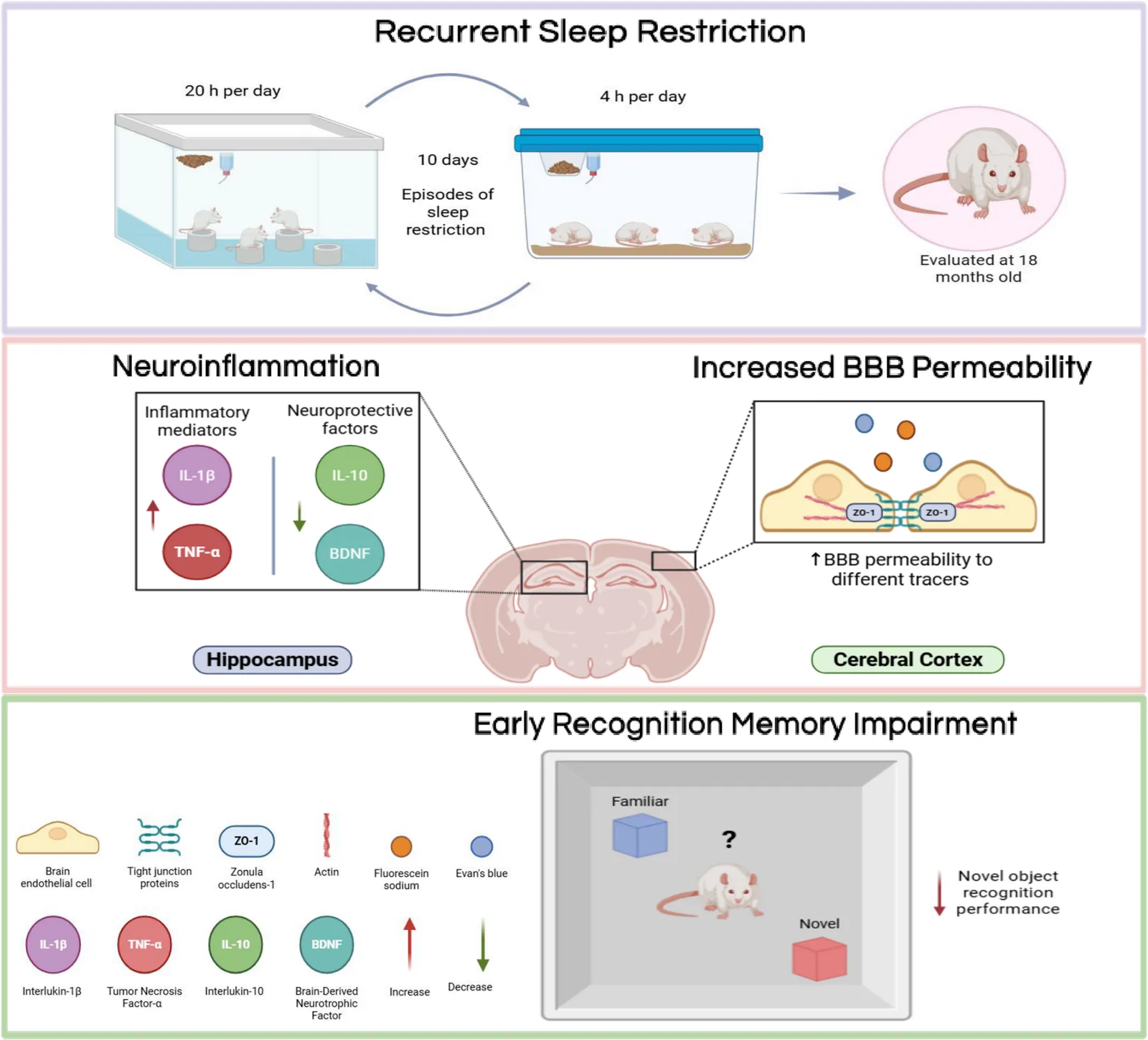

## Introduction

Sleep is essential for maintaining physiological homeostasis through its roles in neurodevelopment, metabolism, immune regulation, brain waste clearance, and cognition [1, 2]. However, insufficient sleep and recurrent sleep restriction have become increasingly common worldwide due to work demands, caregiving responsibilities, shift work, and various lifestyle factors, such as late-night exposure to electronic devices [3–5]. Consequently, recurrent sleep restriction (RSR) has become a major health concern, associated with the development of cardiometabolic disorders like cardiovascular disease, metabolic syndrome, and type 2 diabetes mellitus [6], as well as mood disorders and cognitive impairment [7]. Despite growing evidence linking insufficient sleep to adverse neurological outcomes, whether recurrent sleep restriction contributes to brain aging or modifies age-associated neurobiological processes remains poorly understood.

Women are particularly vulnerable to sleep disturbances, especially during periods of endocrine transition such as perimenopausal and postmenopausal stages [9], as well as due to responsibilities related to child-rearing, household care, or social and occupational factors [9, 10]. Sleep disturbances are highly prevalent among women, and have been associated with poorer quality of life, mood disturbances, cardiometabolic dysfunction, and cognitive decline [11–13]. Despite the high prevalence and clinical relevance of sleep disturbances in women, females remain underrepresented in preclinical sleep research, limiting our understanding of the sex-specific mechanisms by which insufficient sleep influences brain health and aging.

Aging is characterized by progressive changes in brain structure and function, including chronic low-grade inflammation, impaired glymphatic clearance, vascular dysfunction, and progressive cognitive decline [14, 15]. Normal aging also alters sleep architecture and is associated with reduced sleep efficiency, increased sleep fragmentation, and deceased slow-wave sleep [16, 17]. Since both sleep disturbances and aging affect overlapping biological processes, including neuroinflammation, vascular integrity, and waste clearance, recurrent sleep restriction may exacerbate the neurobiological processes associated with brain aging and increase vulnerability to cognitive decline.

Several biological mechanisms have been proposed to explain how recurrent sleep restriction may promote brain dysfunction, including blood-brain barrier (BBB) disruption, neuroinflammation, and cellular senescence. Sleep restriction disrupts BBB integrity by increasing permeability and altering tight junction protein expression [18]. It also induces glial activation and increases the production of pro-inflammatory mediators [19, 20]. Together, these alterations compromise brain homeostasis and may contribute to the decline in learning and memory, functions that are particularly vulnerable during aging [21, 22]. Furthermore, persistent inflammatory states have been proposed to promote cellular changes associated with senescence [20, 23]. Nevertheless, most experimental studies have focused on acute or short-term sleep restriction in young male rodents [20]. Consequently, it remains unknown whether recurrent sleep restriction throughout adulthood induces persistent alterations in BBB integrity, neuroinflammation, cellular senescence, and cognitive function during aging, particularly in females.

Therefore, this study aimed to determine whether recurrent sleep restriction (RSR) exacerbates age-associated alterations in the brain. To this end, we compared aged female rats exposed to recurrent sleep restriction with age-matched females maintained under normal sleep conditions and evaluated BBB permeability, neuroinflammation, markers associated with cellular senescence, and recognition memory, focusing on the cerebral cortex (Cx) and hippocampus (Hc). We found that RSR differentially affected BBB permeability across brain regions, enhanced neuroinflammatory responses, did not modify senescence-associated markers, and was associated with greater impairment of recognition memory in aged females.

## Methods

### Animals

Female Wistar rats (*Rattus norvegicus*) were used in this study. The animals were provided by the animal facility of the Universidad Autonoma Metropolitana, Unidad Iztapalapa. Rats were housed 3–4 per cage in the animal facility under a 12-hour light/dark cycle at 20–22 °C, with food and water available *ad libitum* throughout the study. Animals were allowed to acclimate to the housing conditions for 10 days before the experimental procedures began. The animals’ health status was constantly monitored. A good state of health was considered when the animals did not have tumors, skin or ear infections, and when they ate and drank properly. Rats with tumors and those that went blind were discarded from the study. All animal procedures were strictly carried out according to Mexican Official Ethics Standard NOM-062-ZOO-1999 and the Standard for the disposal of biological waste (NOM-087-ECOL-1995).

### Experimental Groups

Female Wistar rats were allocated into four experimental groups. Young animals were studied at 3 months of age, whereas animals assigned to the aging cohorts entered the study at 6 months of age and were followed until 18 months of age. Allocation to the aging groups was performed by random assignment. The experimental groups were as follows:

1. Young control (Ctrl young, *n* = 15): Three-month-old rats maintained under standard housing conditions and allowed to sleep *ad libitum*.
2. Sleep restricted young (SR young, *n* = 15): Three-month-old rats subjected to 10 consecutive days of sleep restriction, as described below.
3. Aged control (Ctrl old, *n* = 12): Rats maintained under normal sleep conditions from 6 to 18 months of age.
4. Recurrent sleep restriction (RSR old *n* = 14): Rats subjected to repeated 10-day periods of sleep restriction beginning at 6 months of age and repeated every three months until 18 months of age, as described below.

### Sleep Restriction Model

Rats were subjected to sleep restriction using the multiple platform method, as previously reported [24]. Briefly, rats were placed in an acrylic tanks (82 × 59 × 48 cm) containing six platforms (7-cm-diameter) surrounded by water for 20 h per day and where returned to their home cages during the final 4 h of the light phase to allow partial sleep recovery. That protocol was repeated for 10 consecutive days.

To minimize social stress, the animals remained in their cage mates throughout both the sleep restriction and recovery periods (3–4 per cage). In addition, one extra platform was placed inside each chamber to facilitate movement and reduce stress associated with restricted mobility, as previously described [24]. Control animals remained in their home cages under identical environmental conditions and were allowed to sleep ad libitum throughout the experimental period. This protocol has been previously validated by our group and has consistently demonstrated high reproducibility while inducing sustained sleep restriction with minimal mortality and acceptable animal welfare outcomes [18, 20, 24, 25].

### In Vivo Blood-Brain Barrier Permeability Assays

At the end of the experimental protocol, animals (*n* = 4 for all the groups, except for Ctrl old *n* = 3 per group) were deeply anesthetized with sodium pentobarbital (63 mg/kg, i.p). Blood-brain barrier permeability was assessed using a combination of Evans blue (1 mg/mL) and sodium fluorescein (Na-F, 10 mg/mL), which evaluate the extravasation of high- and low-molecular-weight tracers, respectively. Tracers (0.1 mL/20 g body weight) were injected into the left cardiac ventricle and allowed to circulate for 5 min before transcardial perfusion with 0.9% saline for 5 min to remove intravascular dye. The cerebral cortex (Cx) and hippocampus (Hc) were rapidly dissected, weighed, and homogenized in 200 µL of phosphate-buffered saline (PBS, 1×) and 200 µL of absolute methanol to precipitate proteins. Homogenates were centrifuged at 13,500 rpm for 10 min at 4 °C, and 100 µL of the resulting supernatant was transferred to 96-well plates. Na-F (excitation at 485 nm and emission at 535 nm) and Evans blue (excitation at 535 nm and emission at 595 nm) were quantified using a microplate reader (ELISA HLAB; HReader 1). The results are presented as the concentration per unit weight of fresh brain (mg of tracer per g of tissue) as previously described [20, 25].

### Protein Isolation for Western Blot and Enzyme-Linked Immunosorbent Assay (ELISA)

The Cx and the Hc were dissected and homogenized with lysis buffer (20 mM Tris, 0.25 M sucrose, 2 mM EDTA, 10 mM EGTA, 1% Triton X-100) containing a protease inhibitor (cOmplete, 11836153001). The resulting supernatant was aliquoted for Western blot and ELISA analyses. Eight hundred µL of lysis buffer was used for the Cx and 600 µL for the Hc. The tissues were allowed to stand for 20 min. Subsequently, the samples were homogenized under cold conditions and centrifuged at 15,000 rpm for 30 min at 4 °C. The supernatant was collected, and the protein concentration was determined using the Bradford method [26].

### Western Blot

Equal amounts of protein (50 µg per lane) were separated by 10% SDS-PAGE under denaturing conditions and transferred onto PVDF membranes. Each biological sample was analyzed in duplicate by loading two technical replicate lanes. Membranes were blocked for 30 min at room temperature in 10% non-fat dry milk prepared in PBS containing 0.1% Tween-20 (PBS-T). Membranes were then incubated overnight at 4 °C with the following primary antibodies: ZO-1 (Invitrogen, Cat. No. 40–2200, 1:500), occludin (Invitrogen, Cat. No. 40–4700, 1:500), and β-actin (Santa Cruz Biotechnology, Cat. No. sc-69879, 1:2500). After three washes, PVDF membranes were incubated with the secondary antibodies biotinylated anti-rabbit IgG (Vector Laboratories, #BA-1000, 1:2000) and biotinylated anti-mouse IgG (Vector Laboratories, #BA-2000, 1:2000) for 2 h with constant shaking at room temperature. After three more washes, membranes were incubated with an ABC kit (Vectastain PK-6100) for 30 min. Finally, membranes were revealed with a chemiluminescence detection system (Immobilon Western WBKLS0500). Images were acquired using the C-DiGit image generation and analysis system (LI-COR iS image studio, version 6.1).

### Cytokine and BDNF Determination

TNF-α, IL-1β, IL-6, IL-10, and BDNF concentrations were measured using a sandwich ELISA assay, following the manufacturer’s instructions. TNF-α DuoSet ELISA (DY510), IL-1β DuoSet ELISA (DY501), IL-6 DuoSet ELISA (DY506), IL-10 DuoSet ELISA (DY522) and BDNF DuoSet ELISA (DY248), and the DuoSet ELISA auxiliary reagent kit (DY008) were purchased from R&D Systems (Minneapolis, MN, USA). All solutions were prepared using deionized water from a Milli-RQ system (Millipore, MA). Briefly, 96-well plates were coated overnight with the corresponding capture antibody according to the manufacturer’s instructions. After washing, plates were blocked with PBS-Tween 20 (0.05%)/BSA (0.05%) before the addition of tissue homogenates. Brain proteins were incubated for 18 h at 4 °C with PBS-Tween20 (0.05%)/BSA (0.05%), washed three times, and incubated with the corresponding detection antibody for 2 h at room temperature. Bound detection antibodies were detected using the a-HRP system (avidin HRP/streptavidin-HRP, P392056) with TMB/H₂O₂ as the substrate. Optical density readings were performed at 450 nm (Epoch BioTek). All assays were performed in duplicate [26].

### Brain Section

Rats (*n* = 3 per group) were deeply sedated with intraperitoneal sodium pentobarbital (0.063 g/kg body weight) and perfused intracardially for 5 min with 0.9% saline at a flow rate of 10–15 mL/min, followed by a 4% paraformaldehyde solution and 1X PBS at 4 °C for 5 min. The whole brain was extracted and stored in 4% paraformaldehyde at 4 °C for 24 h. The paraformaldehyde was then removed and replaced with a 30% sucrose solution, and the brain was stored at 4 °C. Afterward, the sucrose solution was removed, and the brains were embedded in Tissue Tek (Sakura Finetek, cat. 4583). 30 μm-thick coronal sections were obtained using a Leica CM1850 UV cryostat and mounted on gelatinized slides.

### SA-β-Gal Aactivity Assay

β-Galactosidase enzymatic activity was analyzed following the protocol previously described [26]. Brain sections were washed with PBS 1X, and stained with a solution containing 20 mg/ml of X-gal (V394A, Promega, Madison, WI, United States) in dimethylformamide, 0.2 M citric acid/sodium phosphate buffer pH = 6, 100 mM potassium ferrocyanide, 5 M sodium chloride, and 1 M magnesium chloride. Sections were incubated for 12 h at 37 °C [27].

### Novel Object Recognition Test (NOR)

To determine changes in declarative memory associated with sleep restriction and age, the Novel Object Recognition Test [28] was performed. All tests were conducted during the last 4 h of daylight, from 9:00 a.m. to 13:00. The young control group (Ctrl young) underwent a single test, while the sleep-restricted young group (SR young) underwent the test one day before starting the protocol (from 9:00 a.m. to 13:00 p.m.), and again at the end of the last day of sleep restriction (from 9:00 a.m. to 13:00 p.m.). The older control group (Ctrl old) underwent testing every 3 months, starting at 6 months of age and continuing until 18 months of age. Individuals undergoing a recurrent sleep restriction protocol (RSR) (every three months), were assessed immediately after exiting the protocol (from 9:00 a.m. to 13:00 p.m.). In addition, starting at 12 months of age, they were also assessed before entering sleep restriction at the same time. To discard that the reduced novelty preference index was not driven by motor exhaustion or hypoactivity, the total exploration times were determined.

The NOR test was conducted using a 45 × 45 × 45 cm acrylic box. Immediately after the end of the sleep restriction protocol, rats underwent a 5-min habituation session in the empty testing arena. Twenty-five minutes later, the training phase was performed, during which the animals were allowed to explore two identical objects for 5 min. After another 25-min interval, the testing phase was conducted by replacing one of the familiar objects with a novel object, and the animals were allowed to explore for an additional 5 min. The time spent exploring each object was recorded. After each trial, the acrylic box and the objects were cleaned with 70% ethanol to eliminate olfactory cues. The discrimination index was calculated as the time spent exploring the novel object divided by the total exploration time, multiplied by 100; total exploration time was defined as the exploration time for the familiar object plus the exploration time for the novel object.

### Statistical Analysis

To determine whether the data distribution met the assumption of normality, the data were evaluated using the Shapiro-Wilk test. Subsequently, statistical tests were selected based on the experimental design and the variables analyzed.

For BBB permeability assays, Western blot assays, cytokine and BDNF factor quantification, and the SA-β-gal activity assay, a two-way analysis of variance (ANOVA) was performed, followed by a Tukey post hoc test for multiple comparisons.

To evaluate performance in the NOR test and determine age-related changes, a one-way repeated-measures ANOVA was performed on groups evaluated longitudinally at different ages, followed by a Tukey post hoc test; for comparisons with the young group, a one-way ANOVA followed by a Tukey post hoc test for multiple comparisons was used. To determine changes in behavioral performance following the sleep restriction protocol in longitudinally evaluated groups, a two-way repeated-measures ANOVA was performed, followed by Sidak’s multiple comparisons test; for comparisons with the young group, a two-way ANOVA followed by Sidak’s multiple comparisons test was used. Finally, to evaluate behavioral performance regarding the effect of sleep restriction prior to re-exposure to the protocol in animals evaluated longitudinally at 12, 15, and 18 months of age, a two-way repeated-measures ANOVA was performed, followed by Sidak’s multiple comparisons test.

A p-value of less than 0.05 was considered statistically significant. Statistical analyses were performed using GraphPad Prism (version 8.0.1). Data are presented as mean ± standard error.

## Results

### Recurrent Sleep Restriction Differentially Affects Blood-Brain Barrier Permeability and Tight Junction Protein Expression in the Cerebral Cortex and Hippocampus

**Fig. 1:**
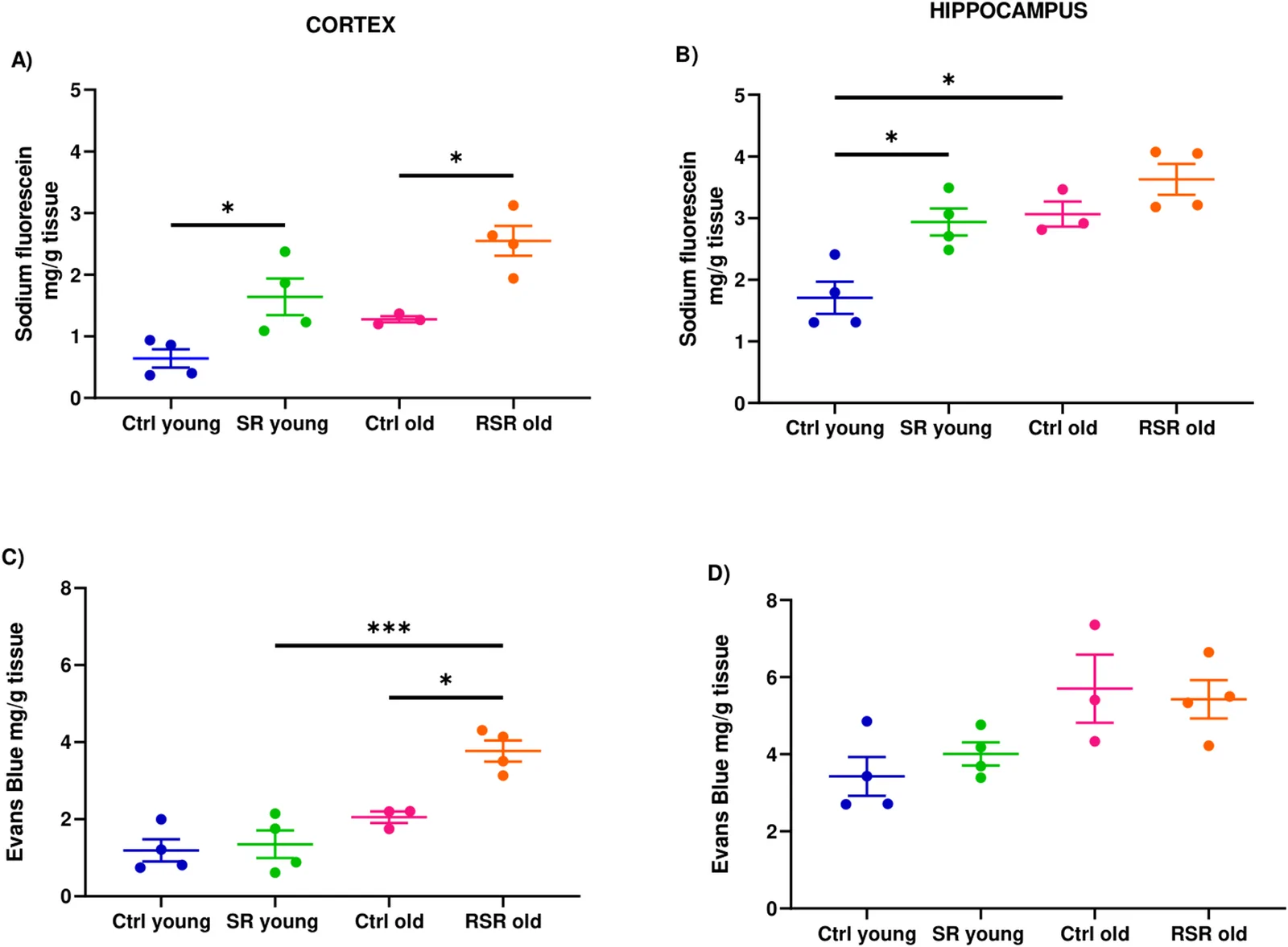
Recurrent sleep restriction increases blood-brain barrier (BBB) permeability during aging. **A** Na-F concentration in the cerebral cortex and **B** in the hippocampus. **C** Evans blue concentration in the cerebral cortex and **D** in the hippocampus. Two-way ANOVA followed by Tukey’s test for multiple comparisons. (*n* = 3 or 4 per group, in duplicate). Data are represented as mean ± SEM. *p < 0.05, **p < 0.001, ***p < 0.0001

Sleep restriction differentially affected BBB permeability depending on the brain region, age, and molecular weight of the tracer (Fig. 1). In the cerebral cortex (Cx), Na-F permeability was significantly increased in the SR young group compared to the Ctrl young group (*p* = 0.0325; Fig. 1A). Likewise, the RSR old group showed a significant increase in Na-F permeability compared to the Ctrl old group (*p* = 0.0112; Fig. 1A), whereas no differences were observed between the Ctrl young group and Ctrl old groups (*p* = 0.2719; Fig. 1A). In the hippocampus (Hc), Na-F permeability was also significantly increased in the SR young group compared to the Ctrl young group (*p* = 0.0151; Fig. 1B), as well as in the Ctrl old group compared to the Ctrl young group (*p* = 0.0132; Fig. 1B). Recurrent sleep loss did not add extra BBB dysfunction to the old rats, as shown by similar BBB permeability to Na-F in Ctrl old and RSR old groups (*p* = 0.4279; Fig. 1B). In contrast, Evans blue permeability was significantly increased only in the Cx of the RSR old group compared to both the SR young and Ctrl old groups (*p* = 0.0004 and *p* = 0.0107, respectively; Fig. 1C). No changes in Evans blue permeability were observed in the Hc under any experimental condition (Fig. 1D).

**Fig. 2:**
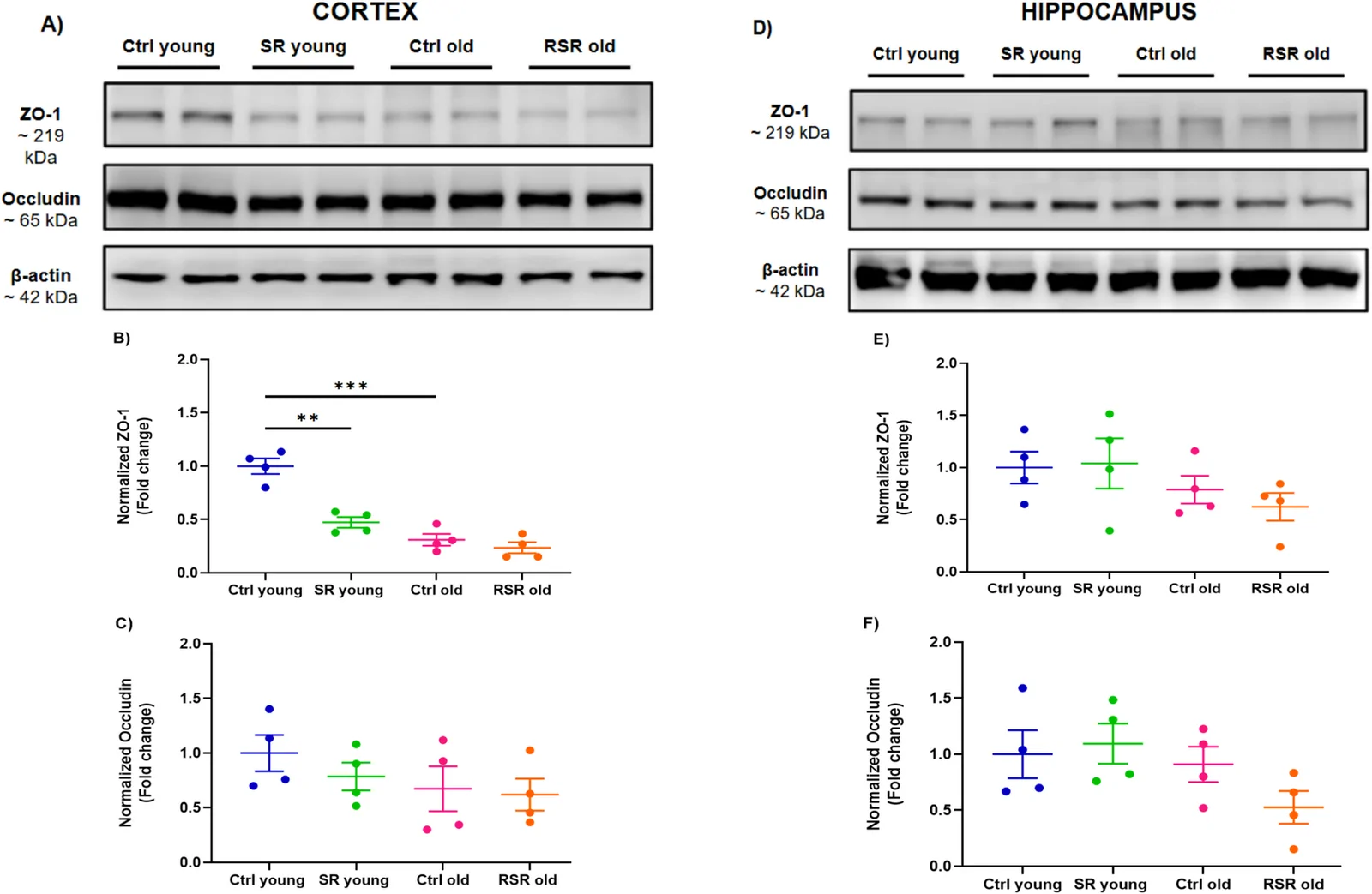
Recurrent sleep restriction decreases levels of the ZO-1 protein in the cerebral cortex during aging. **A** Representative blots of each protein in the cortex using β-actin as a load control. **B** Normalized levels expressed as relative fold change of the ZO-1 and **C** occludin proteins. **D** Representative blots of each protein in the hippocampus using β-actin as a load control. **E** Normalized levels expressed as relative fold change of the ZO-1 and **F** occludin proteins. Data are presented as mean ± SEM. Two-way ANOVA, followed by Tukey’s test for multiple comparisons (*n* = 4 per group, in duplicate). *p < 0.05, **p < 0.001, ***p < 0.0001

To determine whether the changes in BBB permeability were accompanied by alterations in BBB structure, the expression of the tight junction proteins ZO-1 and occludin was evaluated in the Cx and Hc. Sleep loss decreased ZO-1 expression in the Cx of young rats as compared to control rats sleeping *ad libitum (p* = 0.0002, Fig. 2A and B). Downregulation of ZO-1 expression was also observed in the Cx of the Ctrl old groups compared to the Ctrl young group (*p* < 0.0001, Fig. 2A and B). No significant differences in occludin expression levels were observed in either group (Fig. 2A and C).

In the Hc, no significant differences were found in the expression of either ZO-1 (Fig. 2D and E) or occludin (Fig. 2D and F). These findings suggest that the changes in BBB permeability observed in the hippocampus were not accompanied by detectable alterations in the expression of these tight junction proteins.

### Recurrent Sleep Restriction Differentially Alters Inflammatory Cytokine Levels in the Cerebral Cortex and Hippocampus During Aging

Previous work from our research group demonstrated that sleep restriction progressively increases pro-inflammatory cytokine levels in a brain region-dependent manner [20]. Based on these findings, inflammatory mediators were evaluated in the Cx and Hc under the different experimental conditions.

**Fig. 3:**
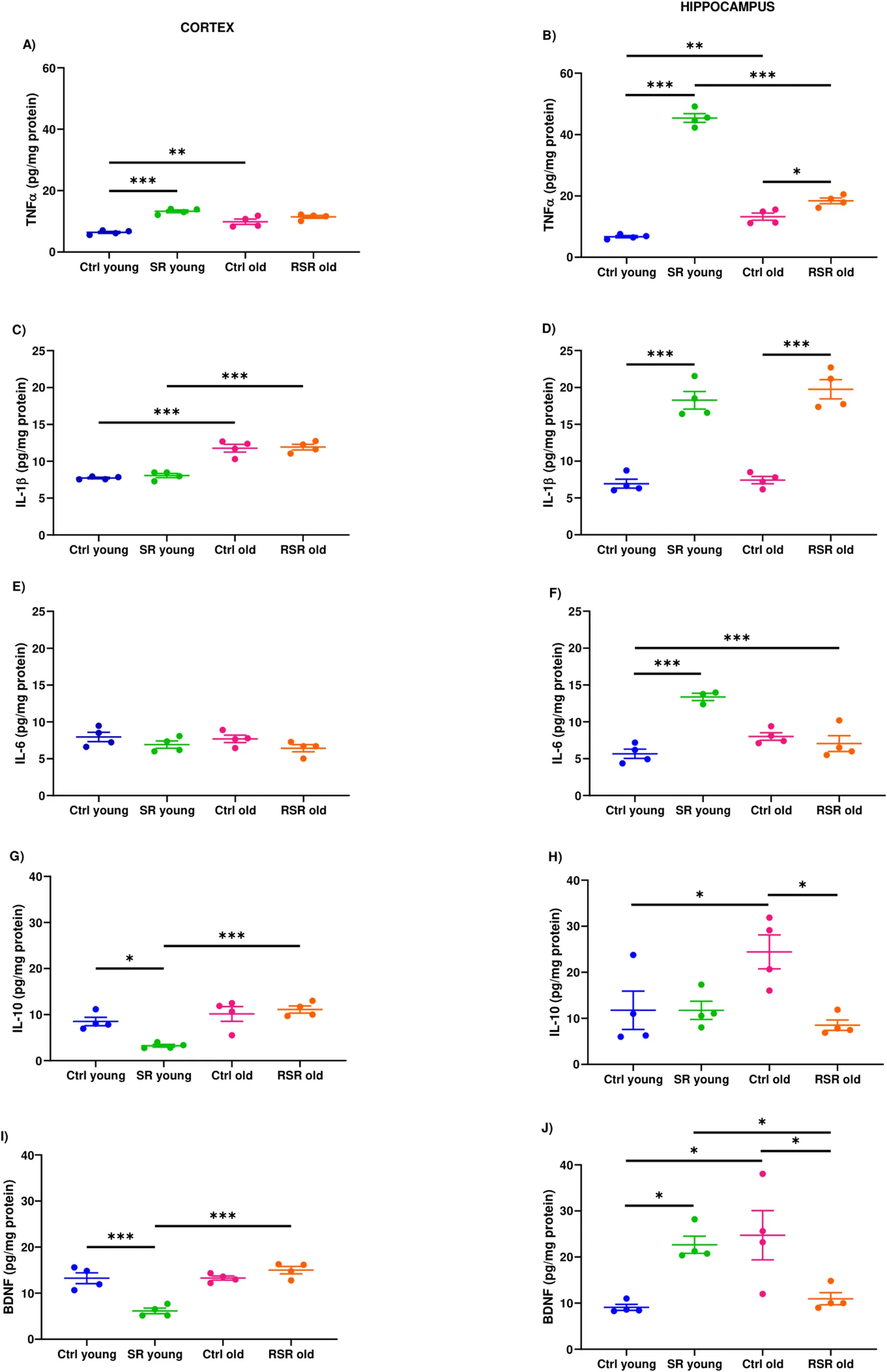
Recurrent sleep restriction increases levels of proinflammatory cytokines in the hippocampus during aging. The graphs show the levels of the proinflammatory cytokines TNF-α (**A**, **B**), IL-1β (**C**, **D**), L-6 (**E**, **F**), IL-10 (**G**, **H**), and BDNF (**I**, **J**) in the cerebral cortex and hippocampus. Two-way ANOVA followed by Tukey’s test for multiple comparisons (*n* = 3 or 4 per group, in duplicate). *p < 0.05, **p < 0.001, ***p < 0.0001. Mean ± SEM

TNF-α concentration significantly increased in the Cx of the SR young group compared to the Ctrl young group (*p* < 0.0001, Fig. 3A). Furthermore, the Ctrl old group showed a significant increase in this cytokine compared to the Ctrl young group (*p* = 0.0044, Fig. 3A). On the other hand, the RSR old group did not increase further the TNF-α concentration compared to the rats of the same age that were not sleep-restricted (*p* = 0.2326, Fig. 3A). In the Hc the highest levels of TNF-α were found in the SR young group compared to the Ctrl young and RSR old groups (*p* < 0.0001, *p* < 0.0001, respectively, Fig. 3B). The Ctrl old group showed a significant increase compared to the Ctrl young group (*p* = 0.0040), while TNF-α concentration in the sleep-restricted aged female group (RSR old) were higher than those in the non-sleep-restricted aged female group (*p* = 0.020, Fig. 3B).

IL-1β concentration significantly increased only in the Cx of the Ctrl old and RSR old groups compared with Ctrl young and SR young groups (both *p* < 0.0001, Fig. 3C). In the Hc, IL-1β levels were significantly higher in SR young and RSR old rats compared to the Ctrl young and Ctrl old groups (both *p* < 0.0001, Fig. 3D). Similar to what was observed with TNF-α, RSR increased IL-1β levels in the Hc of aged rats. No changes in IL-6 levels were observed in the Cx of any group (Fig. 3E), and only a significant increase in this cytokine was found in the Hc of young SR animals compared to the young Ctrl and old CSR groups (*p* = 0.0001, *p* = 0.0007, Fig. 3F).

Regarding the anti-inflammatory cytokine IL-10 levels, a decrease in its concentration was observed only in the Cx of SR young animals compared to the Ctrl young group and the RSR old group (*p* = 0.0139, *p* = 0.0007, respectively, Fig. 3G). In contrast, a significant increase was found in the Hc in the aged females without sleep restriction (Ctrl old) compared to the Ctrl young and RSR old groups (*p* = 0.0487, *p* = 0.0128, Fig. 3H). Similarly, BDNF concentration decreased only in the Cx of SR young females compared to the Ctrl young and RSR old groups (*p* = 0.0002, *p* < 0.0001, Fig. 3I). Interestingly, elevated levels were found in the Hc of the SR young and Ctrl old groups compared to the Ctrl young group (*p* = 0.0295, *p* = 0.0154) and RSR old group (*p* = 0.0447, *p* = 0.0295, Fig. 3J).

### Sleep Restriction Increases the Senescent Associated Activity of the β-Galactosidase Enzyme in the Cerebral Cortex and Hippocampus in Young Rats, But There is No Cumulative Increase in Old Rats After Recurrent Sleep Restriction

Since several of the pro-inflammatory molecules evaluated have been considered markers of the SASP, we performed the SA-β-gal assay to evaluate cellular senescence in those same brain regions.

**Fig. 4:**
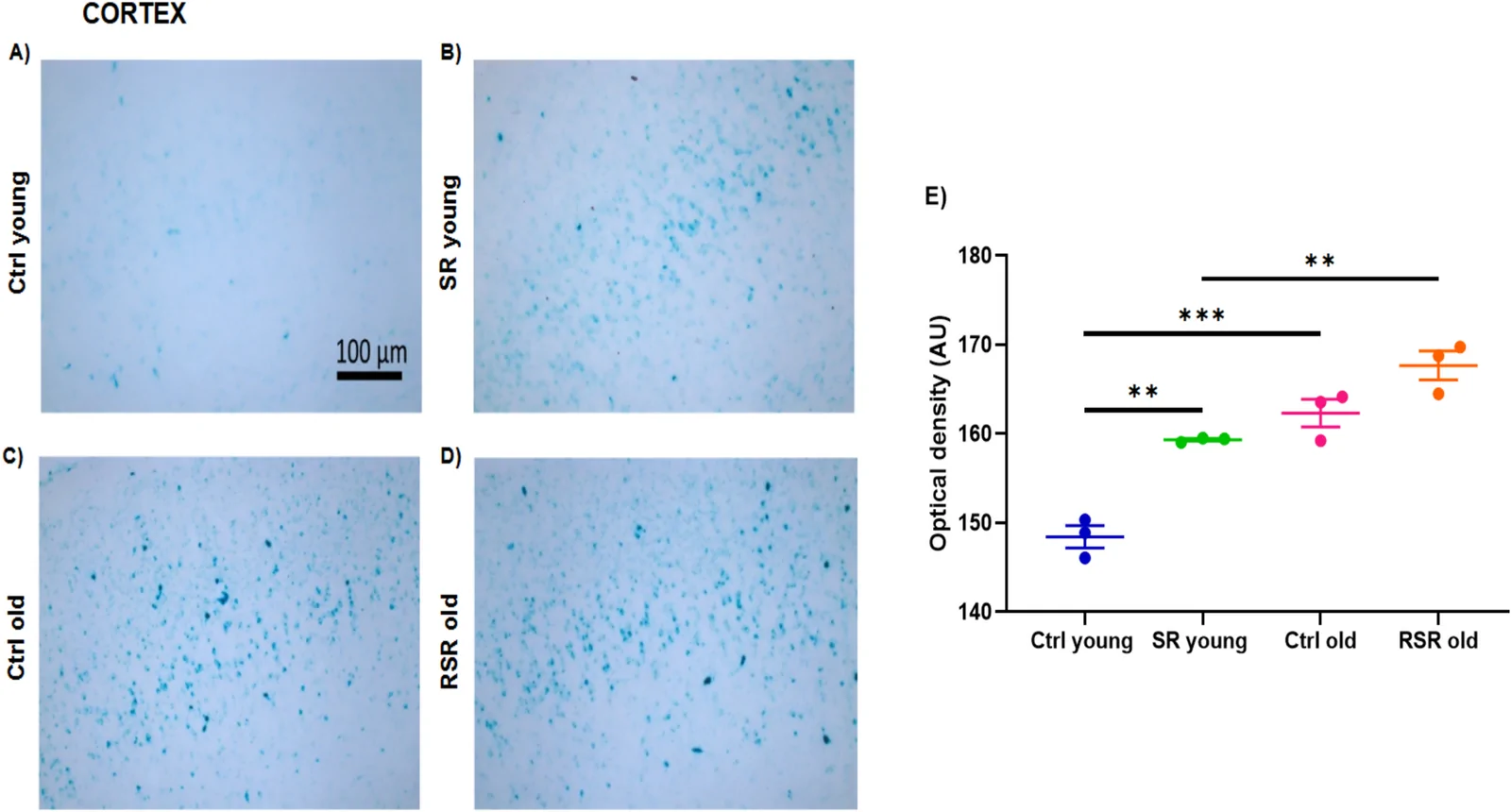
SA β-gal activity in the cerebral cortex during recurrent sleep restriction. Representative images of the cerebral cortex. Bright-field microscopy, 10X objective. **A** Ctrl young, **B** SR young, **C** Ctrl old, **D** RSR old. **E** Optical density in the cerebral cortex. Data are represented as Mean ± SEM. Two-way ANOVA, followed by Tukey’s test for multiple comparisons. (*n* = 3 per group, in triplicate). *p < 0.05, **p < 0.001, ***p < 0.0001

Sleep restriction in young rats significantly increased SA-β-gal optical density in the Cx (SR young vs. Ctrl young, *p* = 0.0015, Fig. 4A and B). Moreover, the Cx of aged females also showed a significant increase in SA-β-gal compared to young females (Ctrl old vs. Ctrl young, *p* = 0.0003, Fig. 4C and D), but recurrent sleep restriction did not increase it further (*p* = 0.0076).

**Fig. 5:**
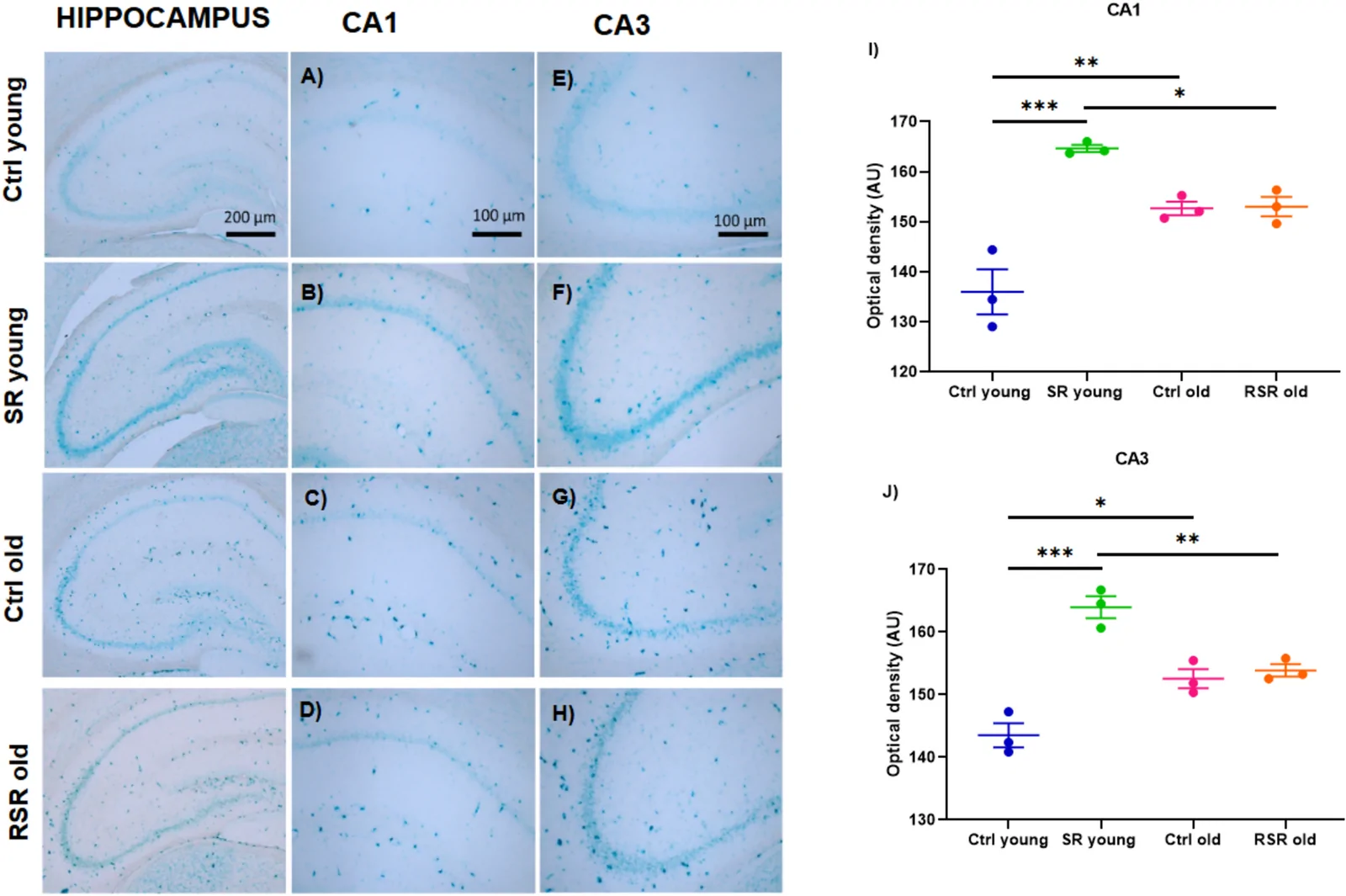
Representative images of the hippocampus. Bright-field microscopy, 5X and 10X objectives. **A**, **E** Ctrl young, **B**, **F** SR young, **C**, **G** Ctrl old, **D**, **H** RSR old, in the CA1 and CA3 regions. **I** Optical density in CA1 and **J** CA3. Data are represented as Mean ± SEM. Two-way ANOVA, followed by Tukey’s test for multiple comparisons. (*n* = 3 per group, in triplicate). *p < 0.05, **p < 0.001, ***p < 0.0001

As it is shown in Fig. 5, sleep restriction increased SA-β-gal in the Hc of young rats. In the CA1 of the SR young group, a significant increase in SA-β-gal was observed compared to all the other groups (Ctrl young *p* = 0.0002, Ctrl old *p* = 0.0435, and RSR old *p* = 0.0496, Fig. 5A–D & I). Likewise, aged females (Ctrl old) showed a significant increase compared to the Ctrl young group (*p* = 0.0075, Fig. 5C and D & I). No significant differences were found between old and old restricted animals. In CA3, the same pattern was observed, Fig. 5E, F, G and H & J (SR young vs. Ctrl young *p* < 0.0001, and RSR old *p* = 0.0089, respectively). The Ctrl old group showed a significant increase with respect to the Ctrl young group (*p* = 0.0162, Fig. 4C, E), and no differences were found between the RSR and the Ctrl old groups (Fig. 5G and H & J).

These data suggest that RSR did not induce a cumulative effect in the Cx and Hc of aging females, being.

### Sleep Restriction Induces Early Deterioration of Declarative Memory

**Fig. 6:**
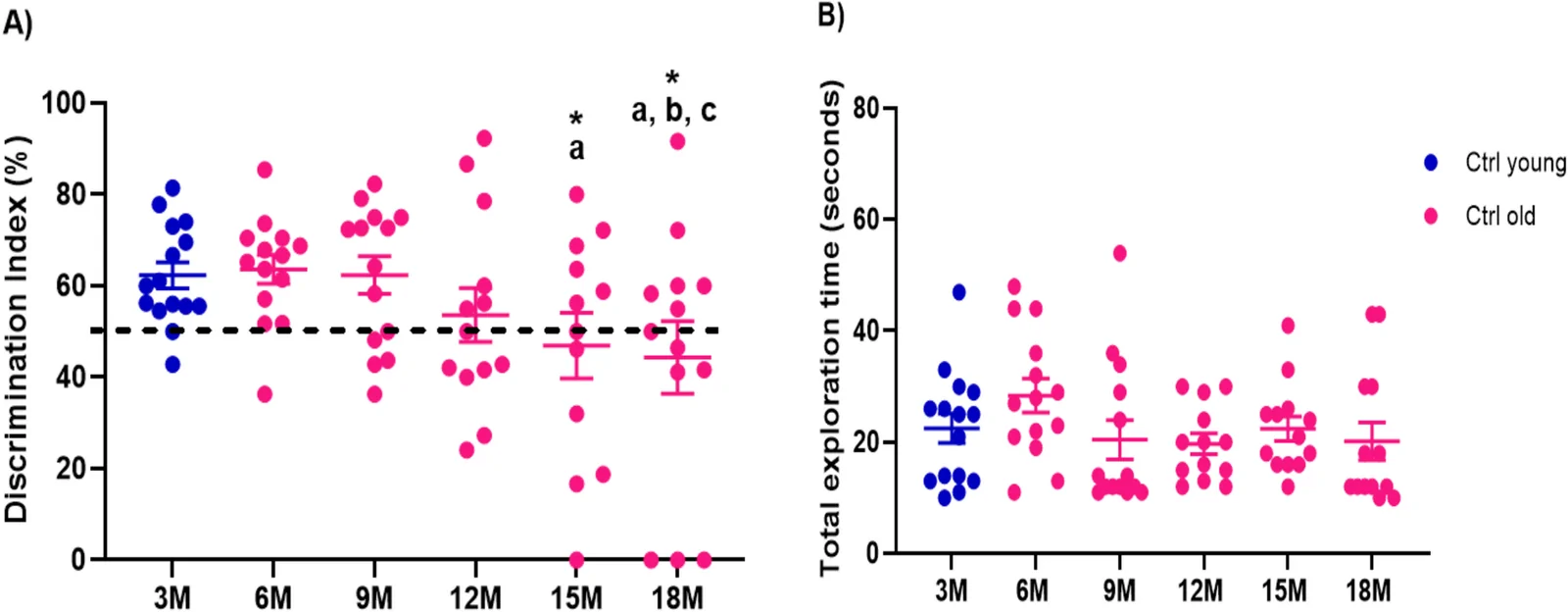
Recurrent sleep restriction induces early cognitive decline. **A** Graphs show the novel object discrimination index for the young control group (blue dots) and the aged control group (pink dots) throughout their lives in the object recognition test. **B** Total exploration time during the test. **C** Graphs show the discrimination index for all experimental groups in the novel object recognition test following the sleep restriction protocol (orange dots). **D** Total exploration time for the experimental groups. **E** Discrimination index in the novel object recognition test for the aged control group (RSR old) prior to the protocol, starting at 12 months of age (purple dots). **F** Total exploration time. (Ctrl young, SR young, *n* = 15; Ctrl old, *n* = 12; RSR old, *n* = 14). Significant difference compared to the Ctrl young group (a); compared to the Ctrl old group at 6 months (b); compared to the Ctrl old group at 9 months (c). * < 0. 05, ** < 0. 001, *** < 0. 0001. Mean ± S.E.M

To determine if these cellular changes were linked to cognitive decline during RSR, recognition memory was assessed using the novel object recognition test. Figure 6A shows a progressive decline in recognition memory during normal aging. This decline became statistically significant from 15 months of age compared with the Ctrl young group (*p* = 0.0420 vas Ctrl young). At 18 months, recognition memory was significantly reduced compared with the Ctrl young group (*p* = 0.0327) and with the Ctrl old group at 6 and 9 months of age (*p* = 0.0330 and *p* = 0.0498, respectively; Fig. 6A).

Additionally, to differentiate the declarative memory performance from physical fatigue, altered locomotor activity, or acute stress the total exploration time was quantified; no significant differences were observed among any of the experimental groups, indicating that the animals exhibited comparable levels of general exploration during the NOR test (Fig. 6B).

As animals aged, greater interindividual variability in the recognition index was observed, suggesting that age-related cognitive decline did not progress uniformly across individuals.

Recognition memory was subsequently evaluated immediately after each sleep restriction protocol from 6 to 18 months of age. As shown in Fig. 6C, acute sleep restriction significantly reduced the recognition index in young rats compared with the Ctrl young group (*p* = 0.0400). A similar pattern was observed in recurrently sleep-restricted aged rats (RSR old). At 6 months, the recognition index was significantly lower than that of the Ctrl young group (*p* = 0.0009, Fig. 6C), but did not differ from that of the SR young group, suggesting that a single sleep restriction episode produced a comparable impairment in recognition memory at 3 and 6 months of age. At 9 and 12 months, RSR old rats continued to exhibit significantly lower recognition indices than Ctrl young animals and age-matched control groups (Fig. 6C), indicating that recurrent sleep restriction accelerated the onset of age-related memory decline.

At 15 months, no significant differences were observed between the RSR old and Ctrl old groups, although both exhibited lower recognition indices than the Ctrl young group (*p* = 0.0420, Fig. 6C). Similarly, at 18 months, the recognition index of the RSR old group remained significantly lower than that of the Ctrl young group and the Ctrl old groups at 6 and 9 months of age (*p* = 0.0002, *p* = 0.0002, and *p* = 0.0017, respectively; Fig. 6C), while no differences were detected between age-matched Ctrl old and RSR old animals. To determine whether the observed changes in the discrimination index were due to differences in exploratory activity following the sleep restriction protocol, total exploration time during the test is shown; no significant differences were observed among any of the groups (Fig. 6D). This suggests that the observed differences in memory performance cannot be attributed to global changes in exploratory behavior.

To determine whether RSR exerted a cumulative effect on recognition memory, the discrimination index was compared before and after each sleep restriction protocol. From 12 months of age onward, no differences were observed between pre- and post-restriction performance (Fig. 6E). Notably, before each sleep restriction protocol, the recognition index was already approximately 50%, indicating chance-level performance and suggesting that the animals were no longer able to discriminate between the familiar and novel objects. Likewise, total exploration time during the test showed no significant differences between the groups (Fig. 6F).

At 15 months, no significant differences were observed between the RSR old and Ctrl old groups, although both exhibited lower recognition indices than the Ctrl young group (*p* = 0.0420, Fig. 6C). Similarly, at 18 months, the recognition index of the RSR old group remained significantly lower than that of the Ctrl young group and the Ctrl old groups at 6 and 9 months of age (*p* = 0.0002, *p* = 0.0002, and *p* = 0.0017, respectively; Fig. 6C), while no differences were detected between age-matched Ctrl old and RSR old animals.

Taken together, these data suggest that, in female rats, sleep restriction did not add a cumulative component to cognitive decline in recognition memory once age-related impairment is established. Instead, RSR appears to accelerate the onset of age-related cognitive decline.

## Discussion

During aging, a progressive decline in brain cellular functions is observed. This phenomenon has been linked to increased inflammation due to processes such as inflammaging and cellular senescence, which are associated with a higher risk of developing cognitive impairment in older adults [29]. Sleep disturbance has recently been proposed as a risk factor for developing age-related diseases like metabolic and neurodegenerative diseases [30]. Moreover, poor sleep quality has been associated with neuroinflammation, glial dysfunction, and alterations in BBB integrity, features commonly observed in neurodegenerative conditions [31]. The impact of insufficient sleep may be particularly pronounced in women, given that sleep disturbances are highly prevalent across most of their lifespan, affecting 40–60% of women, and substantially impair quality of life [8]. Despite this, female animal models, particularly aged females, remain underrepresented in sleep research. Therefore, this study investigated the effects of recurrent sleep restriction during aging in female rats to help address this important knowledge gap.

Our results show that sleep restriction increased BBB permeability to Na-F tracer in the Cx of young animals after 10-days of sleep restriction (Fig. 1A), in agreement with the data reported by He and coworkers using a model of sleep restricted male mice for one week [32]. This group also showed that the permeability loss was reversed if the animals were allowed to sleep normally during 24 h. Previously, we have shown that sleep restriction during 3, 5 and 10 days also impairs BBB function in young male rat and mice [20, 24, 25]. Moreover, the cumulative effect throughout life in the Cx is independent of the effect that could be attributed to the aging process. In the Hc an increase in Na-F tracer permeability was also found in young animals with a single restriction but in old rats changes in hippocampal permeability appeared to be primarily associated with aging rather than RSR.

Gómez-González et al. reported an increase in Evans blue permeability in the Cx of male Wistar rats sleep-restricted for 10 days, which, as in the case above, was restored after 4 h recovery period [24]. Interestingly, in our study, BBB permeability to Evans blue increased only in the Cx of old rats with RSR and not in the young sleep-restricted group, suggesting that repeated periods of sleep restriction along life may compromise the BBB enough to allow the passage of large molecules in the brain parenchyma such as proteins. In addition, our data suggest that the Cx and Hc respond differently to aging and sleep restriction differently to aging and RSR in female rats.

The increased BBB permeability to Na-F and Evans blue in the Cx was accompanied by decreased ZO-1 expression in the SR young and Ctrl old groups, suggesting that both sleep restriction and aging alter the expression of this tight junction protein (Fig. 2A and B). The altered BBB permeability may be mediated, at least in part, by a decrease in ZO-1 expression, a key protein located on the cytosolic side of tight junctions and essential for their organization and function [33]. In contrast, neither sleep restriction nor aging altered total occludin expression in either assessed brain region. One possible explanation is that occludin can be internalized from tight junctions into early and recycling endosomes in response to inflammatory stimuli (e.g., CCL2), without necessarily changing its total protein levels as assessed by Western blot. Thus, BBB dysfunction could be related to a subcellular redistribution of occludin rather than a decrease in its total expression [34]. However, future studies will be necessary to test this hypothesis by examining the subcellular localization of occludin, since the present study assessed only its total protein expression by Western blot.

The former data contrast with previous reports from our group [18, 25], conducted on young male Wistar rats subjected to 10 days of sleep restriction in which increased permeability to Na-F, Evans blue, and rhodamine 123 was observed in both the Cx and Hc. In contrast, the present findings indicate that BBB responses to sleep restriction differ between female and male rats, with a more restricted pattern of permeability changes in females, particularly in the Hc. These results suggest the existence of sex- and region-specific differences in the regulation of BBB integrity during sleep restriction.

Since acute and RSR induce neuroinflammation [20, 35], we evaluated the levels of the proinflammatory cytokines TNF-α, IL-1β and IL-6. In addition to their proinflammatory role, these cytokines are well-recognized components of the senescence-associated secretory phenotype (SASP) secreted by senescent cells during aging and under sleep restriction conditions [20, 36], allowing us to explore the potential contribution of senescence-associated inflammatory pathways.

Our results indicate that both, acute and RSR, increase neuroinflammation in the Hc, as evidenced by increased levels of TNF-α and IL-1β (Fig. 3B and D). This could be associated with the activation of the NLRP3 inflammasome, a complex that regulates IL-1β maturation and secretion. In this regard, it has been reported that in a C57BL/6 male mouse model subjected to sleep restriction (6 h daily for 5 weeks), the CA1 region of the hippocampus shows a significant rise in TNF-α and IL-1β mRNA and protein levels compared to the control group [37]. It is important to mention that pro-inflammatory cytokines in females’ Cx and Hc have been reported to be higher than in males [38]; moreover, it is known that during aging, the rat’s Hc presents significantly higher neuroinflammation compared to the Cx, leading to the well-known age-related cognitive decline [39] and concurring with our results.

On the other hand, the differential neuroinflammatory responses observed in both regions could be mediated by the involvement of glial cells, such as microglia; these cells possess greater morphological complexity and phagocytic capacity in the Hc compared to the Cx [40]. Furthermore, age-dependent, brain-region-specific microglial heterogeneity has been demonstrated; in 2-month-old mice, microglia in the Hc and medial prefrontal Cx exhibited greater total length and a higher number of microglial process intersections, whereas in 20-month-old mice, a markedly larger microglial size, reduced total microglial process length, and reduced maximum length was observed. After 48 h of sleep deprivation, microglia density decreased in aged mice, whereas they increased in young mice [41]. Suggesting that sleep deprivation may dysregulate microglial homeostasis in a region- and age-dependent manner. Here we aimed to determine whether RSR in female rats accelerates BBB alterations, neuroinflammation, and memory impairment during aging development. Therefore, we did not evaluate microglial activation or its role in this progression. Additionally, it is known that astrocytes, both senescent and reactive, contribute to neuroinflammation [36] so future research should consider evaluating glial involvement in RSR.

Interestingly, in our study, the increase in neuroinflammation was not associated with increased blood-brain barrier (BBB) permeability. Therefore, it is possible that hippocampal neuroinflammation and BBB dysfunction do not occur simultaneously during sleep restriction. A plausible explanation is that inflammatory responses are initiated before detectable alterations in BBB permeability arise. Our recent work supports this hypothesis since neuroinflammatory changes were detected during the early stages of sleep restriction and preceded the progressive deterioration of BBB integrity in young mice [20]. In that study, brain levels of IL-1β remained significantly elevated throughout the 10-day sleep restriction protocol, but their increase was detected as early as day 5 in the Cx, and day 3 in the Hc [20]. Another study showed that when humans were sleep restricted for five days, the plasma levels of inflammatory cytokines such as IL-6, TNF-α, and IFN-γ, did not increase [43]. Taken together, these findings are consistent with the possibility that RSR initiates the inflammatory response within the brain, independently of a detectable peripheral inflammatory response. Sustained neuroinflammatory signaling could subsequently contribute to BBB dysfunction by affecting tight junction proteins and increasing paracellular permeability. Likewise, early microglial activation in response to the accumulation of metabolic waste during insufficient sleep could contribute to the observed cytokine profile [44].

However, it should be noted that this early neuroinflammation does not necessarily rule out an initial systemic inflammatory response; moreover, the temporal relationship and directionality between neuroinflammation and BBB dysfunction were not directly assessed in the present study and remain to be experimentally determined. Future longitudinal studies should clarify the sequence of these events and determine the consequences of hippocampal neuroinflammation under RSR conditions. Furthermore, it is interesting to notice that during aging, the inflammatory response differed between Ctrl and recurrently sleep-restricted aged rats, which could reflect the dysregulation of the immune system response known as inflammaging [45].

The response of the anti-inflammatory and trophic factors, such as IL-10 and BDNF, varied according to age and acute or recurrent sleep restriction. During aging, female rats exhibited increased levels of both IL-10 and BDNF; however, this response was attenuated following RSR (Fig. 3H and J). These findings are consistent with previous reports describing increased hippocampal BDNF levels during physiological aging, but not under conditions of pathological aging [46]. Together, these observations raise the possibility that RSR interferes with compensatory mechanisms that normally accompany healthy brain aging.

The differential responses observed between males and females under sleep-restricted conditions could be partly explained by the action of female sex hormones. Estrogens are known to have a protective role for synaptic function [47], inflammation [48], cognition [49], and BBB integrity [50]. Hence, one possibility is that the female hormonal environment might contribute to the differential regulation of the BBB, neuroinflammation, and BDNF expression during sleep restriction described in the present study. However, this possibility should be addressed experimentally in future studies.

To determine the role of cellular senescence in these conditions, in addition to the proinflammatory cytokines, we analyzed the activity of senescence-associated β-galactosidase, a marker of increased lysosomal content and activity [51]. Our results indicate that RSR does not exacerbate this process in aged female rats, as no significant differences were observed in the Cx or Hc compared to the age-matched control groups (Fig. 4A, C). It is possible that the senescent cell load has reached a peak with age, thereby limiting the detection of further changes under RSR. Moreover, it remains to be determined which cell types in the central nervous system undergo senescence under these conditions. Future studies incorporating additional senescence markers (e.g., p16, p21, or SASP components) together with cell-type-specific markers will be necessary to confirm the senescent phenotype and identify the cellular populations involved. Furthermore, it cannot be ruled out that, during advanced stages of aging, other cellular mechanisms, such as apoptosis, necroptosis, or pyroptosis [52–54], become more relevant, although these were not evaluated here.

Taken together, the observed changes in BBB permeability, tight junction protein regulation, neuroinflammatory profile, and IL-10/BDNF levels prompted us to evaluate whether these altered mechanisms were accompanied by cognitive impairment. Using the novel object recognition test, we found that sleep restriction impaired recognition memory during the early stages of the aging process. Rats subjected to repeated sleep restriction exhibited reduced discrimination indexes at 6, 9, and 12 months of age compared with age-matched controls, whereas control animals at these ages still performed similarly to young controls (Fig. 6C). By 15 and 18 months of age, however, the discrimination index of the aged control group had declined to levels comparable to those of the sleep-restricted group, and no significant differences were detected between them. These findings suggest that repeated sleep restriction accelerates the onset of age-related memory impairment rather than producing an additional detectable decline at later ages.

A limitation of our study was that the behavioral tests were conducted during the light phase, immediately following the sleep restriction protocol, which may have affected performance uniformly, especially in older animals, as it coincided with their rest phase. Even so, no differences in exploratory activity during the test across any of the experimental conditions were observed. Furthermore, the object recognition paradigm itself may have limited sensitivity for detecting additional impairment in advanced stages of aging. Once animals exhibit recognition rates close to chance (~ 50%), a floor effect may occur, making it difficult to distinguish between age-related impairment and a possible cumulative effect of sleep restriction. In this regard, one suggestion would be to use more sensitive paradigms, such as the L-maze (V-maze), where it is possible to determine the number of errors, etc. [55]. An additional limitation of the present study is the absence of a group of aged rats (18 months) subjected only to acute sleep restriction. The inclusion of this group would have allowed a more precise determination of whether the observed changes in BBB integrity, neuroinflammation, and IL-10/BDNF levels result from repeated sleep restriction throughout aging or represent the response to a single episode of sleep restriction in late life. Therefore, future studies should incorporate this experimental group to better distinguish the effects of acute versus repeated sleep restriction in the aging brain.

## Conclusion

Overall, our findings demonstrate that the impact of sleep restriction varies across the aging process. Acute sleep restriction in young female rats induced widespread BBB dysfunction, neuroinflammation, increased senescence-associated activity, and memory impairment, whereas RSR during aging produced more selective alterations, primarily affecting cortical BBB permeability and hippocampal inflammatory status without further increasing senescence-associated β-gal activity. RSR was associated with an earlier onset of recognition memory impairment, however, once age-related cognitive deficits became established, no additional differences could be detected using the novel object recognition task. Taken together, these findings contribute to our understanding of the harmful impact of recurrent sleep restriction and underscore the need for future studies to better define the underlying mechanism of this phenomenon throughout the lifespan in relation to brain aging in women.

## Data Availability

No datasets were generated or analysed during the current study.

## Abbreviations

BBB: Blood-brain barrier
BDNF: Brain-derived neurotrophic factor
CCL2: Chemokine monocyte chemoattractant protein-1
Cx: Cerebral cortex
ELISA: Enzyme-linked immunosorbent assay
Hc: Hippocampus
IL-1β: Interleukin-1 beta
IL-6: Interleukin-6
IL-10: Interleukin-10
Na-F: Fluorescein-sodium
NOR: Novel object recognition test
RSR: Recurrent sleep restriction
SASP: Senescence associated secretory phenotype
SA-β-gal: Senescence-associated β-galactosidase
TNF-α: Tumor nuclear factor alpha
ZO-1: Zonula occludens 1
